# Combined ascorbic acid and T_3_ produce better healing compared to bone marrow mesenchymal stem cells in an Achilles tendon injury rat model: a proof of concept study

**DOI:** 10.1186/s13018-019-1098-9

**Published:** 2019-02-18

**Authors:** Francesco Oliva, Nicola Maffulli, Clarissa Gissi, Francesca Veronesi, Lucia Calciano, Milena Fini, Silvia Brogini, Marialucia Gallorini, Cristina Antonetti Lamorgese Passeri, Roberta Bernardini, Rosella Cicconi, Maurizio Mattei, Anna Concetta Berardi

**Affiliations:** 1 Department of Orthopaedics and Traumatology, Surgery and Dentistry, Azienda Ospedaliera San Giovanni di Dio e Ruggi d’Aragona, University of Salerno School of Medicine, Salerno, Italy; 2 Centre for Sports and Exercise Medicine, Queen Mary University of London, Barts and the London School of Medicine and Dentistry, Mile End Hospital, London, UK; 3 Institute of Science and Technology in Medicine, Keele University Medical School, Stoke on Trent, UK; 4 U.O.C. of Immunohaematology and Transfusion Medicine, Laboratory of Stem Cells, Spirito Santo Hospital, Pescara, Italy; 5 Laboratory of Preclinical and Surgical Studies, Research Innovation and Technology Department (RIT), IRCCS Rizzoli Orthopedic Institute, Via di Barbiano 1/10, 40136 Bologna, Italy; 6 Dipartimento di Sanità Pubblica e Medicina di Comunità, Sezione di Epidemiologia e Statistica Medica, Università di Verona, 37134 Verona, Italy; 7 Department of Medical, Oral and Biotechnological Sciences, University “G. d’Annunzio” Chieti-Pescara, Chieti, Italy; 8 Interdepartmental Service Centre - Station for Animal Technology, University of Rome “Tor Vergata”, Rome, Italy; 9 Department of Biology, University of Rome “Tor Vergata”, Rome, Italy

**Keywords:** Ascorbic acid, Thyroid hormones, T_3_, bone marrow mesenchymal stem cells, Tendon

## Abstract

**Background:**

This pilot study aimed to ascertain whether the local application of ascorbic acid (AA), of T_3_, and of rat (r) bone marrow mesenchymal stem cells (BMSCs), alone or in all possible combinations, promoted healing after an Achilles tendon injury in a rat model.

**Methods:**

An Achilles tendon defect was produced in 24 6–8-week-old male inbred Lewis rats. The animals were then randomly divided into eight groups of three rats each. The tendon defect was filled with 50 μL of phosphate-buffered saline (PBS) containing (1) 50 μg/mL AA (AA group), (2) 10^−7^ M T_3_ (T_3_ group), (3) 4 × 10^6^ rBMSCs (rBMSC group), (4) 50 μg/mL AA + 10^−7^ M T_3_ (AA + T_3_ group), (5) 4 × 10^6^ rBMSCs + 50 μg/mL AA (rBMSC + AA group), (6) 4 × 10^6^ rBMSCs + 10^−7^ M T_3_ (rBMSC + T_3_ group), (7) 4 × 10^6^ rBMSCS + 50 μg/mL AA + 10^−7^ M T_3_ (rBMSC + AA + T_3_ group), and (8) PBS only (control group: CTRL). All treatments were administered by local injection immediately after the tendons had been damaged; additionally, AA was injected also on the second and fourth day from the first injection (for groups 1, 4, 5, and 7), and T_3_ was injected again every day for 4 days (for groups 2, 4, 6, and 7). At 30 days from initial treatment, tendon samples were harvested, and the quality of tendon repair was evaluated using histological and histomorphological analysis. The structure and morphology of the injured Achilles tendons were evaluated using the modified Svensson, Soslowsky, and Cook score, and the collagen type I and III ratio was calculated.

**Results:**

The group treated with AA combined with T_3_ displayed the lowest Svensson, Soslowsky, and Cook total score value of all tissue sections at histopathological examination, with fiber structure close to regular orientation, normal-like tendon vasculature, and no cartilage formation. AA + T_3_ also showed the highest collagen I and the lowest collagen III values compared to all other treatments including the CTRL.

**Conclusion:**

There are potential benefits using a combination of AA and T_3_ to accelerate tendon healing.

## Introduction

About 3 to 5 million patients worldwide experience tendon injuries each year [1]. Tendon healing is slow and often incomplete: the currently available techniques for surgical intervention and repair are inadequate [2–5]. New procedures should be developed to produce better outcome. In this context, tissue engineering (the combination of cells, growth factors, and carriers into functional tissue) is a promising therapeutic option in tendon regenerative medicine.

Recently, cell-based, and especially stem cell-based, strategies for tendon regeneration have attracted enormous attention. Bone marrow mesenchymal stem cells (BMSCs) have been extensively studied, displaying tenogenic differentiation capacity [6], and are currently the most widely used type of stem cell [7, 8], since they have potential for improving tendon repair [6], accelerating healing, and regenerating normal tissue. At present, however, BMSCs alone seem ineffective [9, 10], and recent biological approaches, such as BMSCs in combination with growth factors, and specialized delivery systems are being explored to enhance tendon healing [11–13].

Our previous reports have shown that tendons express thyroid hormone (TH) receptor isoforms and that T_3_ and T_4_ hormones enhance tenocyte proliferation in vitro [14, 15]. Moreover, T_3_ and T_4_ contrast apoptosis in healthy tenocytes in a dose- and time-dependent manner [14, 15]. THs (especially T_3_) stimulate cellular proliferation and type I collagen formation, the major fibrillar collagen in tendons. Furthermore, we have shown that, in the presence of ascorbic acid (AA), T_3_ increases collagen I expression, and additionally, T_3_ also increases ECM protein secretion in the presence of AA and, in particular, biglycan and COMP (cartilage oligomeric matrix protein) expression [14, 16, 17]. Robinson et al. recently demonstrated that biglycan is required for mechanical proprieties in the tendon and for collagen fiber realignment after loading [18]. COMP is known to co-localize with collagen I [19], and a marked reduction of COMP in tendon injury has recently been demonstrated, suggesting the possibility of its use as a marker for tendon injury [20]. In addition, AA on its own triggers the proliferation of tendon-derived cells [16, 17]. AA also decreases nitric oxide synthesis (NOS) in the same experimental condition. NOS inhibition exerts beneficial effects on tendon regeneration and function in a murine of Achilles tendon rupture [21]. When AA is combined with T_3_, it synergistically induces an increase in tenocyte proliferation [17].

The role of thyroid hormones in stem cell function is still unclear and further study is required. Previous investigations have shown that the thyroid hormone T_3_ exerts a dose-dependent effect on the chondrogenic and osteogenic differentiation of BMSC in female rats, promoting chondrogenic matrix formation and collagen synthesis respectively [22, 23]. T_3_ treatment increases the differentiation of BMSCs induced to cardiomyocytes and promotes their maturation [24]. The combined treatment of BMSC with exercise and thyroid hormones enhances new neuronal cell generation and attenuates apoptosis in ischemia strokes in mice [25].

There is some uncertainty as to whether BMSC transplantation alone is sufficient to achieve satisfactory outcomes in tendon repair and healing. Therefore, it is necessary to assess the effects of combining other therapies with cell therapy to increase their therapeutic efficacy in tendon injury. In trying to translate all these findings into clinical practice, it would be reasonable, first of all, to hypothesize that AA and T_3_ in combination would be beneficial to human tendon healing in vivo. Moreover, no studies have yet been performed to understand the influence of thyroid hormones on BMSCs in tendon regeneration, nor of thyroid hormones and BMSCs in combination with AA.

Therefore, the present pilot study, using a rat Achilles tendon injury model, aimed to verify the roles of rBMSC, AA, and T_3_ in promoting healing after local inoculation at the site of injury. rBMSC, AA, and T_3_ were used alone and in every possible combination and compared with an untreated control. After a period of 30 days, the efficacy of local administration of all combinations of rBMSCs, AA, and T_3_ on healing was evaluated.

## Materials and methods

### Ethics

All the experiments were conducted according to the protocols of good animal experimentation under the Italian Health Ministry approval n°513/2016-PR and in accordance with international laws and policies (Directive 2010/63/EU of the European Parliament and of the Council, Italian Legislative Decree 26/2014, *Guide for the Care and Use of Laboratory Animals*, NIH publication 85-23, 1985).

### Isolation, culture, and characterization of rat BMSCs

rBMSCs were isolated from the bone marrow of the tibia and femur of three 6–8-week-old male inbred Lewis rats (ENVIGO, Wyton, UK), as previously described [26]. The bone marrow cells were seeded into a tissue culture flask in MesenCult Basal Medium (STEMCELL Technologies, Vancouver, Canada), supplemented with penicillin/streptomycin (100 U/mL–100 μg/mL) and 10% FBS, and incubated at 37 °C, in a 5% CO_2_ atmosphere with 95% relative humidity. Three days after seeding, floating cells were removed, and the medium was replaced with a fresh medium. Adherent cells were allowed to reach approximately 80% confluence (12–17 days for the first passage). Cells were detached following treatment with StemPro accutase (ThermoFischer Scientific, USA) and replaced every 6–8 days at approximately 80% confluence. Cells at second passage (P2) were used unless otherwise stated. When the cells in the culture flasks were nearly confluent, the cells in the primary culture were sub-cultured and collected for the study. In addition, mesenchymal stem cells (MSCs) were tested by flow cytometry (Becton Dickinson, Franklin Lakes, New Jersey, USA) using specific surface markers, being negative for CD45 and positive for CD90 [27]. Debris particles were excluded from the analysis by gating on forward and side scatter (FSC and SSC) morphological parameters. Dead cells were gated out by propidium iodide (0.07 μg/mL, Sigma-Aldrich, Saint Louis, USA) staining; 10.000 viable and non-debris events in the morphological gate were recorded for each sample. Cells were acquired on a CytoFLEX flow Cytometer (Beckman Dickinson, CA, USA). The analysis was performed using CytExpert Software (Beckman Dickinson). The stem cell characteristics of bone MSCs were routinely confirmed by their trilineage differentiation abilities (adipocytes, osteoblasts, and chondrocytes) when cultured under appropriate conditions in vitro before being used for experimental procedures as previously described [27, 28]. Adipocytes were visualized by Oil Red-O staining, osteoblasts evidenced by alizarin red, and chondrocytes were visualized with Alcian Blue. Isolated rBMSCs (P0) were cryopreserved according to standard procedures in liquid nitrogen in 90% fetal bovine serum + 10% DMSO and stored in liquid nitrogen in vials until transplantation.

### Animal surgery and pilot study design

Twenty-four 6–8-week-old male inbred Lewis rats (ENVIGO) were housed under controlled conditions in the Interdepartmental Service Centre - Station for Animal Technology, University of Rome “Tor Vergata” (Italy), supplied with standard diets (4RF18; Mucedola srl, Italy) and water ad libitum. Under general anesthesia induced using an intramuscular injection of tiletamine/zolazepam (50 mg/kg) (Zoletil 100, Virbac Italia SRL, Italy) and xylazine (10 mg/kg) (Rompun Bayer AG, Germany), the right Achilles tendon was laid open by a skin incision approximately 10-mm long on the medial aspect of the right hind limb of each rat. In each animal, an Achilles tendon defect 2 mm in diameter was produced as previously described [29] with some modifications. Briefly, the Achilles tendon was separated from the surrounding tissue, and the medial, more prominent component of the Achilles tendon, the *m. flexor digitorum superficialis*, was isolated. Using a sterile punch (diameter 2 mm), which was placed around the Achilles tendon at a distance of approximately 2 mm from the calcaneus, a full-thickness hole, 2 mm in diameter, was made. The skin was closed by a continuous suture using absorbable 4.0 absorbable monofilament sutures. The animals were divided into eight groups (with three rats per group). The injured tendon was filled with 50 μL of phosphate-buffered saline (PBS), a physiological buffer solution which contained (1) 50 μg/mL AA (AA group), (2) 10^−7^ M T_3_ (T_3_ group), (3) 4 × 10^6^ rBMSCs (rBMSC group), (4) 50 μg/mL AA + 10^−7^ M T_3_ (AA + T_3_ group), (5) 4 × 10^6^ rBMSCs + 50 μg/mL AA (rBMSC + AA group), (6) 4 × 10^6^ rBMSCs + 10^−7^ M T_3_ (rBMSC + T_3_ group), (7) 4 × 10^6^ rBMSCS + 50 μg/mL AA + 10^−7^ M T_3_ (rBMSC + AA + T_3_ group), and (8) PBS only (control group: CTRL). Additionally, AA was injected again on the second and fourth day following the initial injection (for groups 1, 4, 5, and 7), while T_3_ was injected again every day for 4 days (for groups 2, 4, 6, and 7). Post-operatively, antibiotics and analgesics were administered: 0.5 mL/kg Baytril (Bayer AG, Germany) and 0.1 mL/kg/day Rimadyl (Veterfarma SpA, Italy). After 30 days, the animals were anesthetized and then euthanized using CO_2_ in specially designed chambers. Tendons were explanted, embedded in optimal cutting temperature (O.C.T.) compound medium (Sakura Finetek USA, Inc., Torrance, CA, USA), and quickly frozen in liquid nitrogen-cooled isopentane for sectioning at a thickness of 12 μm using a Leica (Leica Biosystems, Wetzlar, Germany) cryostat, and then stained with hematoxylin and eosin or Picro-Sirius red solution for histological and histomorphometric analyses and examined under white light and polarized light microscopy, respectively. Sections were examined independently by two pathologists in a blinded, random order.

### Histological and histomorphometric examination

For histological and histomorphometric analyses, three sections per animal and three animals per group were evaluated for each experimental group. Three sections of each specimen were placed onto one slide at an approximate distance of 250 μm. Six areas were selected from each section to cover the entire surface of the tendon and were collected using an optical microscope (BX51, Olympus Italia Srl, Milano, Italy) at × 10 magnification, coupled to Aperio ScanScope (Aperio ScanScope CS, Aperio Technologies, Leica Biosystems, USA) digital image analysis system. A modified, semi-quantitative score obtained by the three different scores of Soslowsky, Svensson, and Cook’s scoring systems was used (Table 1) [30–32]. These scores take into account four different parameters: fiber structure, cellularity, vascularity, and cartilage formation. Each parameter was scored on a 4-point scale of 0–3, as follows: 0, normal; 1, slightly abnormal; 2, abnormal; and 3, markedly abnormal. The samples were scored according to whether there was a significant abnormal appearance. The total score is the sum of the values of each parameter varied between 0 (normal tendon) and 12 (severest abnormality) [33]. For the validation study, pathologists were additionally asked to make overall examinations for each specimen. The specimens were anonymized and randomized by an IRCCS Rizzoli Orthopedic Institute employee not involved in the study. For polarization microscopy, each section (three sections per animal, three animals per group) were imaged using × 8 magnification. To prevent variations in illumination and magnification, all pictures were recorded by the same person. The sections underwent further evaluation using computerized image analysis software (Aperio Area Quantification FL Algorithm). Color intensity and color amount (i.e., positively stained area) were measured by Aperio ScanScope CS (Aperio Area Quantification FL Algorithm, Aperio ScanScope CS Leica Biosystems). Collagen type I and type III percentages were calculated by these automated measurement of the red-orange fiber area (collagen type I) and pale green fiber area (collagen type III) from Picro-Sirius red staining [34].

**Table 1:** Semi-quantitative histomorphometric scoring system as modified by Svensson, Soslowsky, and Cook

Parameter	Grade 0	Grade 1	Grade 2	Grade 3
Fiber structure, Svenson and Solowsky scores	Normal parallel collagen fibers	Mild changes (< 25% slightly separated fibers)	Moderate changes (25–50% disorganized, separated, and deteriorated fibers)	Marked changes (> 50% disorganized and hyalinized fibers)
Cellularity (aspect), Cook scores	Elongated nuclei and absence of cytosol	Oval nucleus and absence of cytosol	Round nucleus and little cytosol	Round nucleus and abundant cytosol
Vascularity, Svensson score	Few vessels, parallel to the fiber	Slight increase of vessels	Moderate increase of vessels	Marked increase of vessels
Cartilage formation	No cartilage	Isolated cartilage nodules	Moderate cartilage formation (25–50%)	Extensive cartilage formation (> 50%)

### Statistical analysis

Data are expressed as mean values ± standard deviation (SD). Statistical analysis was performed using the STATA v.14.2 software with data reported at a statistical significance level of *p* < 0.05. Two-level regression models (group-treatment = level 1 unit; rat = level 2 unit) with a random intercept term at level 2 and the treatment group as fixed effects, followed by adjusted Bonferroni post hoc test, were used to compare histomorphometric results (total score and collagen ratio). The estimates of the fixed and random parameters were obtained by using the restricted maximum likelihood method. The denominator degrees of freedom of the null sampling distributions of test statistics for fixed effects were computed by using the repeated method to take the bias associated with the small sample into account.

## Results

### Identification of rBMSC

**Fig. 1:**
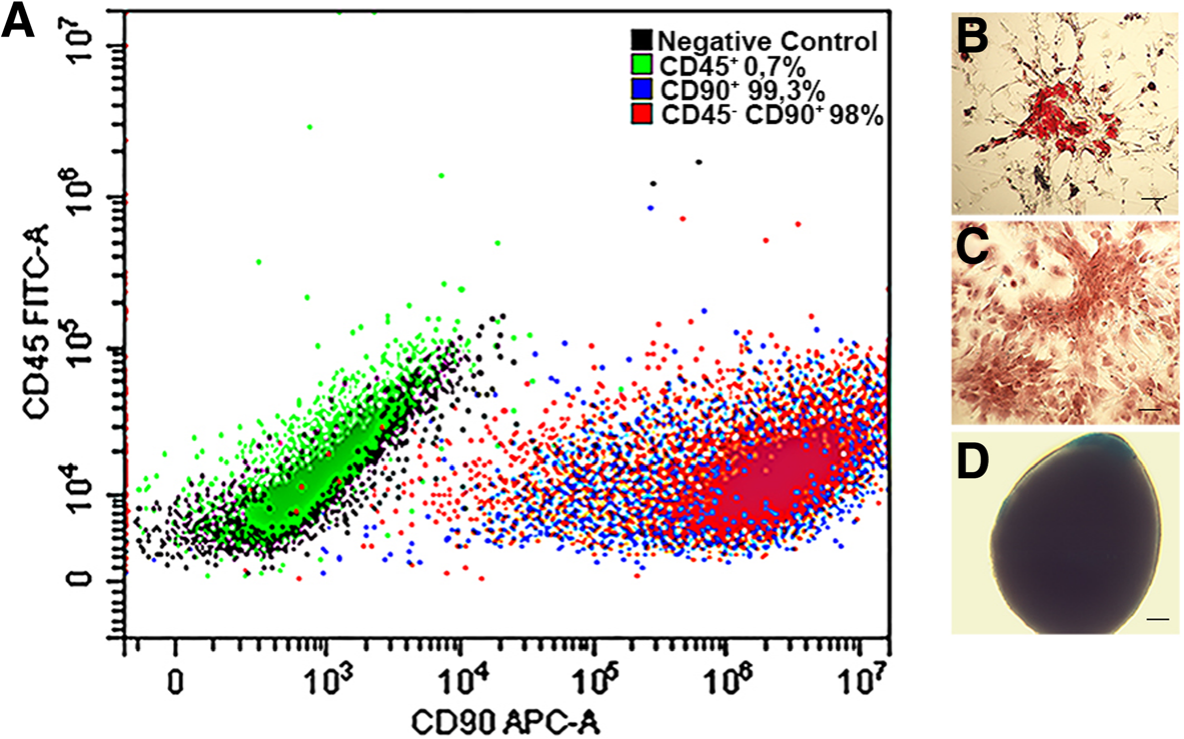
rBMSC characterization. An example of rBMSCs phenotype and functional analysis. **a** Flow cytometry analysis of MSCs for surface markers (CD45^+^ isotype control, CD90^+^ isotype control, negative control) showed that isolated bone marrow-derived cells expressed CD90, but not the hematopoietic marker CD45, confirming that only mesenchymal cells had been isolated (CD45^−^CD90^+^ cells population). **b**–**d** In vitro differentiation of mesenchymal cells: the cells were incubated in lineage-specific induction media and then analyzed by histochemical and cytological staining for **b** adipocytes (Oil Red-O staining), **c** osteoblasts (alizarin red staining), and **d** chondrocytes (Alcian Blue staining)

Once they had been isolated and cultured, the rBMSCs were analyzed for immunophenotype by flow cytometry, which confirmed the expression of CD90 and the lack of CD45 (Fig. 1a). In addition, rBMSCs were functionally characterized by differentiation into adipocytes, osteoblast, and chondrocytes (Fig. 1b–d), displaying the typical characteristics of MSCs [35].

### Local injection of AA + T_3_ ameliorated tendon repair

**Fig. 2:**
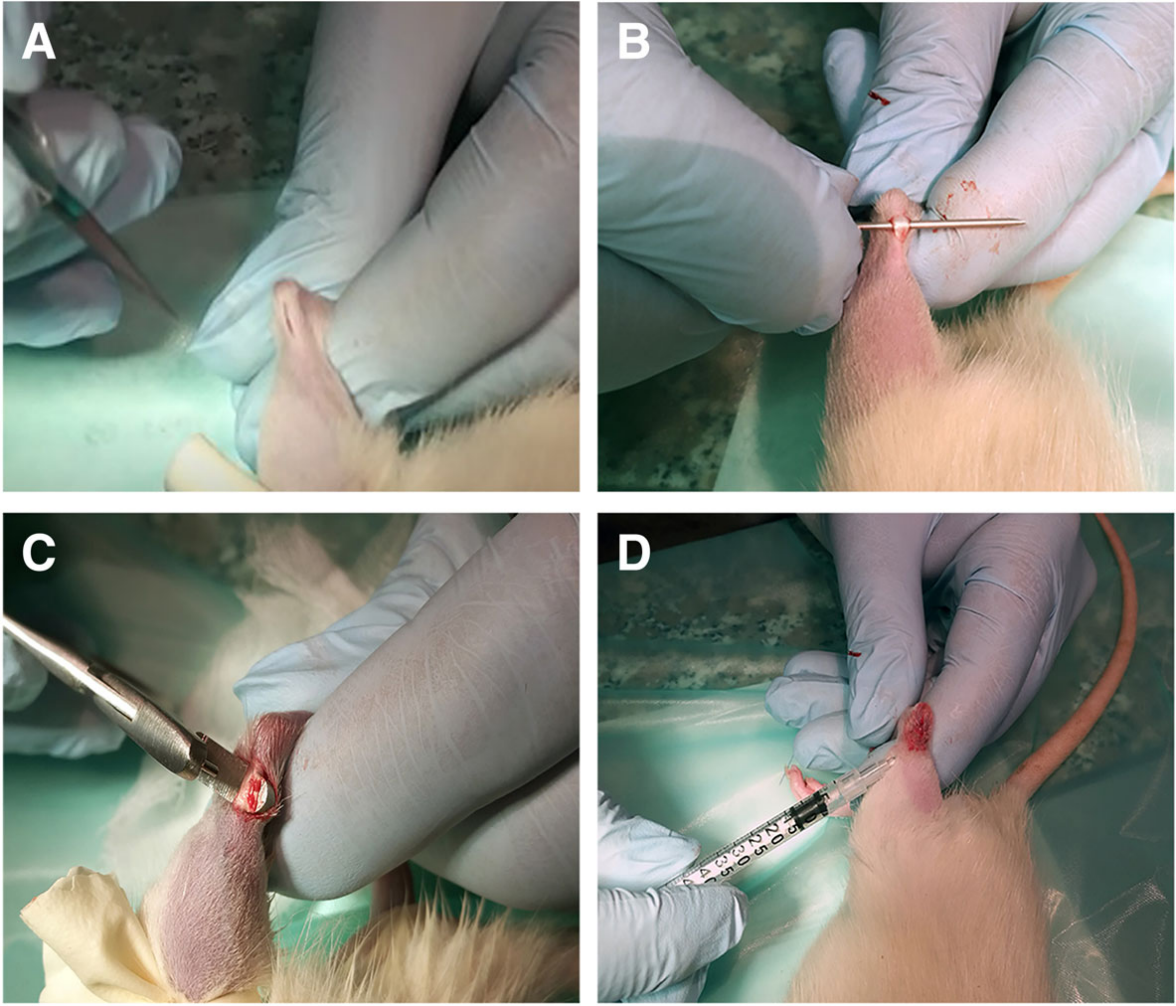
Surgical procedure. **a**, **b** Under general anesthesia, the right Achilles tendon was laid open by a skin incision approximately 10-mm long on the medial aspect of the right hind limb. **c** A 1-mm sterile punch was placed around the Achilles tendon at a distance of approximately 2 mm from the calcaneal bone. **d** Treated area: the skin was closed by a continuous suture and each treatment locally micro-injected

**Fig. 3:**
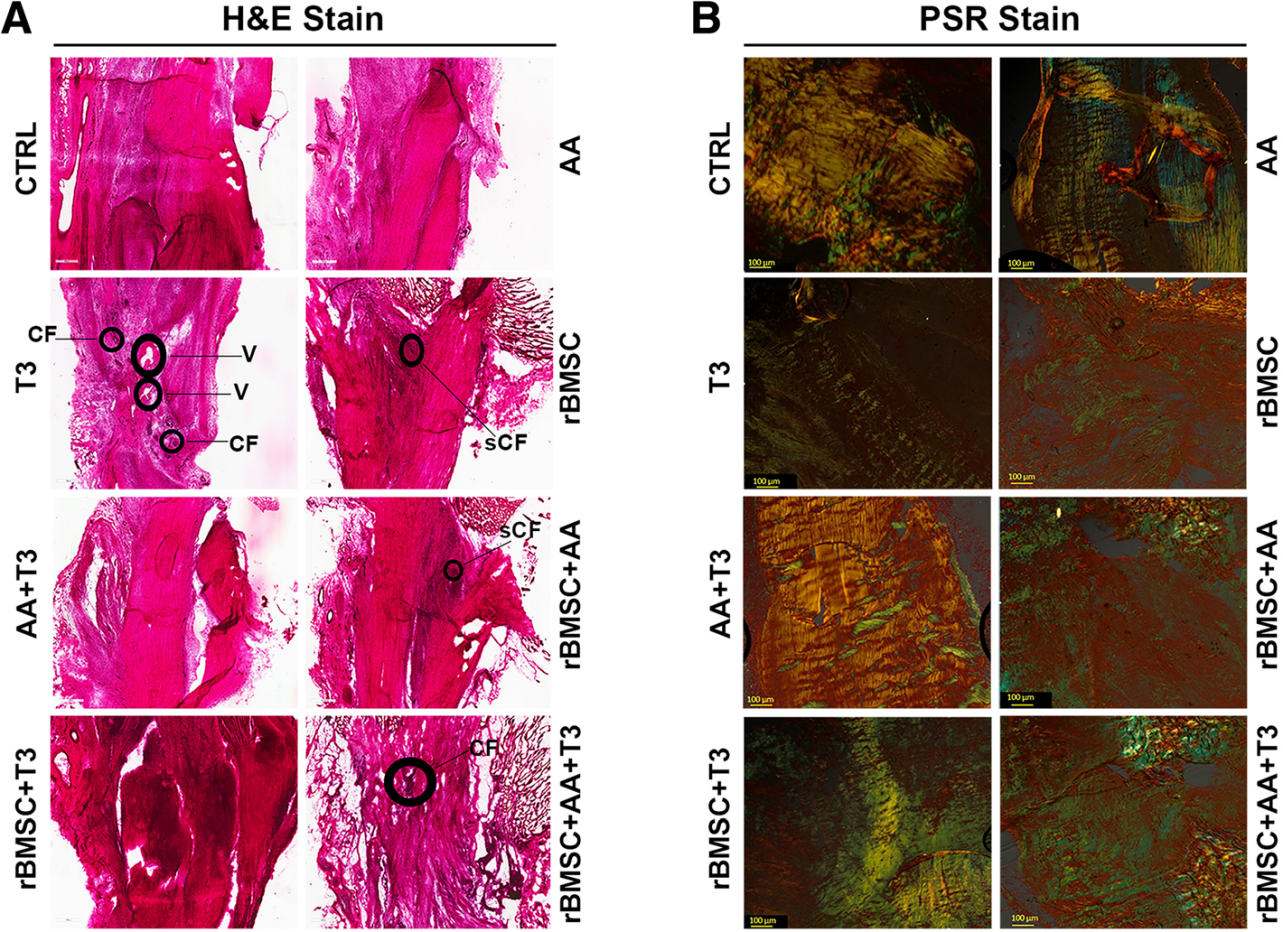
Histological analysis of tendon healing at 30 days from injury/start of treatment. **a** The injured Achilles tendons were then stained with H&E for each type of treatment, and the quality of tendon repair was evaluated based on properties displayed. [CF (cartilage formation), V (vessel); _S_CF (slight cartilage f)] (× 500). **b** longitudinal sections of the right hind limb rat Achilles tendons stained by Picro-Sirius red staining (PSR): (magnification × 4, scale bar = 500 μm). The thicker, mature collagen fibers appear red-orange (type I collagen); the thinner collagen fibers appear pale green (type III collagen)

Surgical procedures and treatments (Fig. 2) did not determine any additional accidental damage and no clinical signs of necrosis or tissue infection. At gross examination post mortem, scar tissue was visible at the site of the core lesion in the center of each tendon. Macroscopically, no differences were visible between the groups. No macroscopic signs of necrosis or tendon degeneration were observed. The paratenon covering the tendon was of normal thickness, as were the collagen fibers of the epitenon, and no inflammatory infiltrate or other morphological changes were seen. Intra-class correlation for histology scores was excellent for inter-observer reliability (0.79–0.97 depending on score/sub-score). The lesions could be identified clearly on H&E staining. Histological analysis indicated incomplete restoration of structural integrity (Fig. 3).

**Fig. 4:**
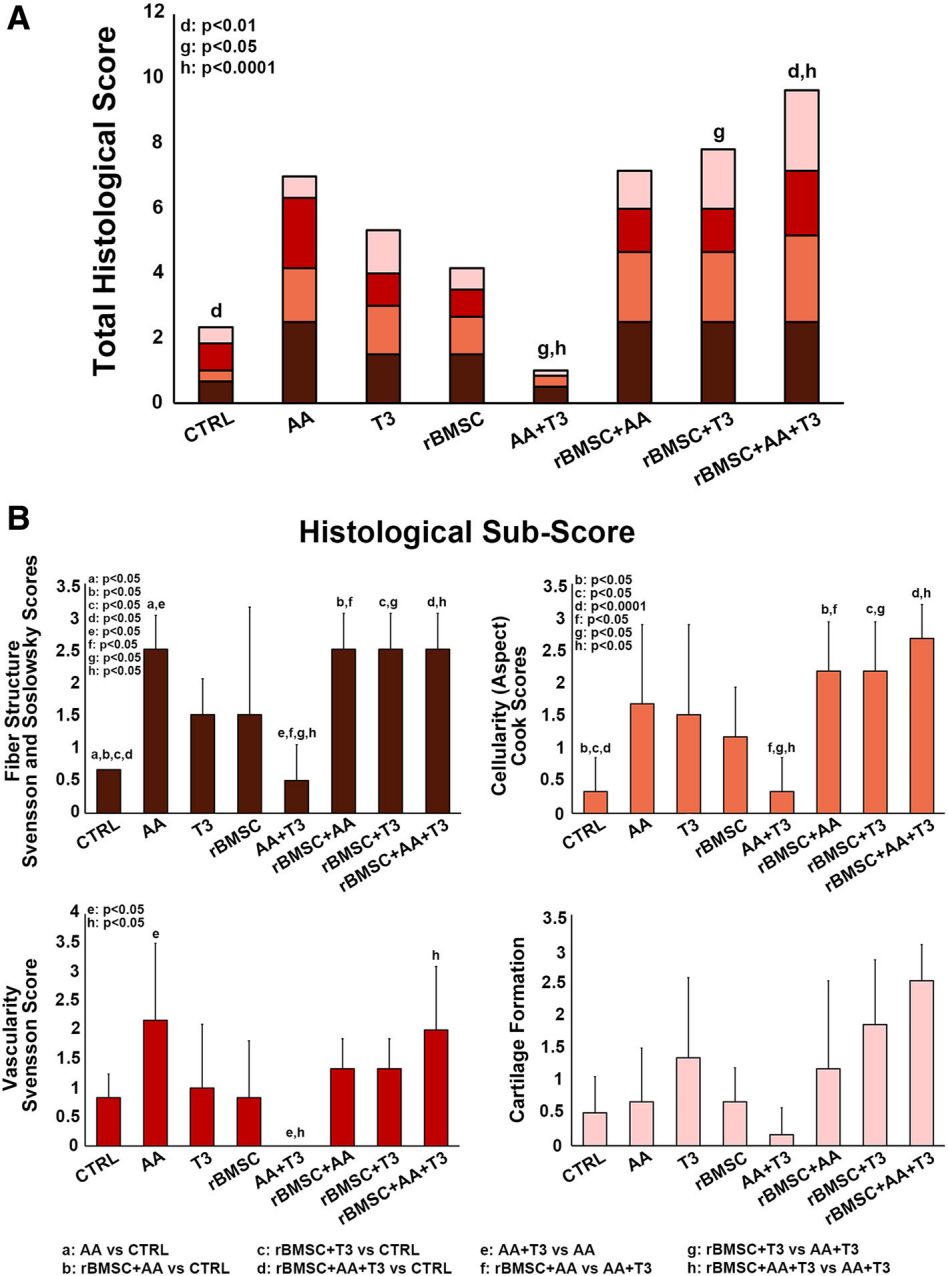
Stacked histograms of the semi-quantitative histomorphometric score for treated and untreated control groups. **a** Total score for healthy tendon score, including cartilage formation, vascularity, cell aspect, and fiber structure scores (mean, *n* = 3). **b** Detailed sub-scores. One-way ANOVA test and adjusted Bonferroni (two-way) post hoc test

Semi-quantitative histomorphometric results are reported in Fig. 4. The total histological score was lower in the AA + T_3_-treated group (score 1) than in all the other group treatments (AA score 7; T_3_ score 5.3; rBMSC score 4.16; rBMSC + AA score 7.16; rBMSC + T_3_ score 7.16; rBMSC + T_3_ + AA score 9.66) including the CTRL group (score 2.3) (Fig. 4a). Specifically, in the group treated with AA + T_3_, score values were lower for fiber structure (close to regular orientation, score 0.5) than those in the CTRL group (0.7); vasculature (vessels run inconspicuous coursing parallel to the collagen fiber bundles in the septa, like in a normal tendon, score 0) than those in the CTRL group (score 0.8); and cartilage formation (slight cartilage formation was only observed in one section, score 0.1) than those in the CTRL group (slight formation of cartilage in three sections for one of the three animals, and in two sections for the second of the three animals, score 0.5). The rBMSC group had the same histology score as the CTRL group for cartilage formation (slight cartilage formation was observed in two of the three sections for the first animal and in one section only for the other two animals in the group) (Fig. 4b). The AA + T_3_ group total scores were significantly lower than those of the rBMSC + T_3_ group (*p* < 0.005) and the rBMSC + AA + T_3_ group (*p* < 0.0001). The rBMSC + AA + T_3_ group scored significantly higher than the CTRL group. Specifically, the AA + T_3_ group sub-scores were lower for fiber structure in comparison with the AA group, the rBMSC + AA group, the rBMSC + T_3_ group, and the rBMSC + AA + T_3_ group (*p* < 0.005); for cellularity scores, the AA + T_3_ group sub-scores were lower than for all other groups and, most significantly, than for the rBMSC + AA group, the rBMSC + T_3_ group, and the rBMSC + AA + T_3_ group (*p* < 0.005); for vascularity, the AA + T_3_ group sub-scores were significantly lower than for the AA group and the rBMSC + AA + T_3_ group (*p* < 0.005), and the AA + T_3_ group cartilage formation sub-scores were lower than all the other group treatments (Fig. 4b). In the AA + T_3_ group, the vascularity sub-score value was similar to that of a healthy tendon (Fig. 4b), and the value was significantly lower than that for the AA + T_3_ group and for the rBMSC + AA + T_3_ group (*p* < 0.005) (Fig. 4b).

**Fig. 5:**
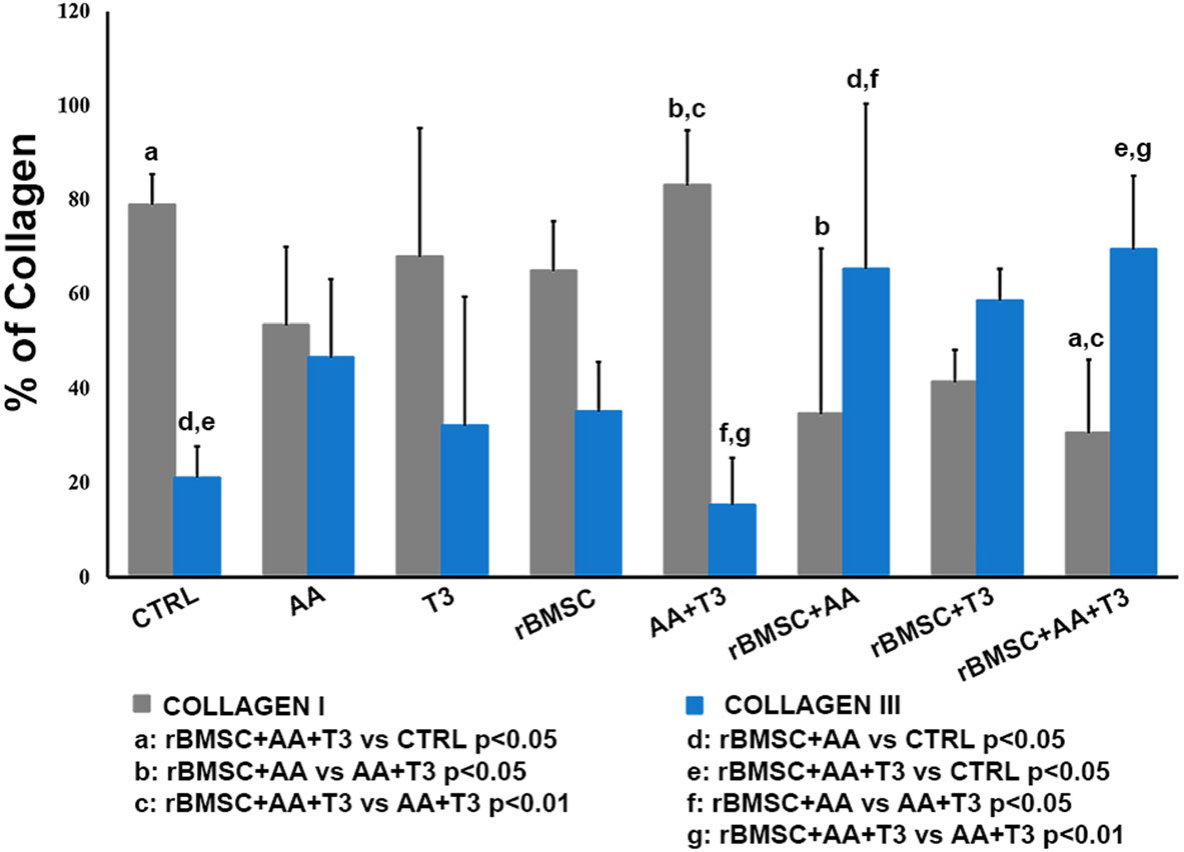
Histograms of type I and type III collagen content, expressed in percentages, for treated and untreated control groups (mean ± SD, *n* = 3). One-way ANOVA test and adjusted Bonferroni (two-way) post hoc test

Analysis of the collagen ratio revealed that the association AA + T_3_ exerted greater effect on tendon healing than all the other treatments. Specifically, the collagen type I value was significantly higher than in the rBMSC + AA group and the rBMSC + AA + T_3_ group (*p* < 0.05; *p* < 0.01); the AA + T_3_ group collagen type III value was significantly lower than in the rBMSC + AA group and the rBMSC + AA + T_3_ group (*p* < 0.05; *p* < 0.01) (Fig. 5). The collagen type I value of the rBMSC + AA + T_3_ group was significantly lower than the CTRL (*p* < 0.05), and the collagen type III value was significantly higher in the rBMSC+AA+T_3_ group than the CTRL group (*p* < 0.05) (Fig. 5).

## Discussion

We wished to test the effect of local micro-injection of AA, T_3_, and rBMSCs, individually and in every possible combination, on tendon healing in damaged rat tendons. Our investigation logically follows prior studies which demonstrated the relevance of ascorbic acid and T_3_ in combination with the biological function of tenocytes. Novel preliminary findings demonstrate the efficacy of ascorbic acid in combination with the T_3_ hormone in improving tendon healing, confirming our previous in vitro studies [14, 16, 17]. Histological evaluation confirmed better restoration of normal tendon architecture with an optimal alignment of tendon fibers and blood vessels, although the continued presence of high cellularity suggests that the regenerative process was not complete. Furthermore, in the tendons treated with AA + T_3_, the analysis of the collagen ratios demonstrated a higher expression of collagen type I, and lower expression of collagen type III, than in all the other groups, including the CTRL group. Comparison of the AA + T_3_ treatment with the AA alone, and the T_3_ alone, showed that the co-administration of AA and T_3_ yielded greater beneficial effects than the administration of either separately. The histological score values and the collagen ratios were worse for AA alone, and for T_3_ alone, than for the CTRL group, although they were better than for the rBMSC + AA group, the rBMSC + T_3_ group, and the rBMSC + AA + T_3_ group. This was not the case for the histological score value of the rBMSC group. The healing process of an injured tendon passes through three main phases, featuring distinctive cellular and molecular cascades: (i) inflammatory; (ii) reparative (proliferation), characterized by cellularity and matrix production, which is mostly collagen type III; and (iii) remodeling (consolidation and maturation), replacing collagen type III with collagen type I and fiber organization [36]. Taken together, the results indicate that ascorbic acid acts in synergy with T_3_ to accelerate tendon healing.

Our results agree not only with our own previous data, but also with other studies [3, 37]. Ascorbic acid stimulates a wide range of tissue functions; it contributes not only to increased collagen synthesis and cross-linking, but also plays key roles in stem cell function, such as in the differentiation of mesenchymal stem cells into tenocytes [37, 38]. Supplementation with ascorbic acid also enhances tendon healing in mouse models [39]. Moreover, thyroid hormones are major determinants of tissue function, playing well-defined roles in cell signaling. The role of thyroid hormones in tenocyte physiology is a novel area of study in this field [14, 15, 40, 41]. Moreover, THs regulate the function of stem cells, controlling self-renewal, proliferation, and differentiation. In vivo studies suggest high intracellular level requirement of T_3_ to support normal myogenesis and muscle repair after cardiotoxin injury [42]. All the animals in the groups treated with rBMSC healed less well than all the other groups, including the CTRL group, when analyzed histopathologically and histomorphologically. The group which received rBMSC only showed better results than the rBMSC + AA group, the rBMSC + T_3_ group, and the rBMSC + AA + T_3_ group. At present, the use of mesenchymal stem cells in tendon healing is still the subject of controversy, debate, and study. Indeed, only three BMSC-based therapies have shown favorable outcomes in rat studies with improved histological and biomechanical tendon properties [43]. However, in accordance with our investigation, several other studies were not able to demonstrate beneficial effects of BMSC on tendon healing. Furthermore, no difference in collagen production or in extracellular matrix organization has been demonstrated. The source of stem cells for implantation is still under debate. In the present investigation, the cells used were derived from the bone marrow, but recent developments suggest that stem cells derived from the tendons themselves show the greatest promise to improve healing [44]. Moreover, it should be underlined that growth factors in combination with the cells have recently been shown to require controlled spatiotemporal delivery to the repair site to improve tendon healing [12, 44]. Consequently, greater enhancement of tendon healing could be obtained by specialized delivery approaches, for example, by tissue engineering using a variety of scaffolds [12, 44]. The exact mechanisms of tendon healing and the precise roles that different cell types play in the process are, as yet, not clear, and further studies are necessary to develop a range of suitable techniques involving stem cells to favor and hasten tendon regeneration and repair [45].

Our results do not confirm the findings of previous studies regarding the use of AA in combination with MSCs [39, 46]. This is likely because of the different methodology and different model used and the different source of the stem cells. Kang et al. used AA to stimulate MSCs before transplantation in a mouse tendonitis model, using adipose-derived cells [39]. Durant et al. used an in vitro model employing tendon-derived stem cells, which may have had some bearing on the results obtained [46]. T_3_ has not been used in previous in vivo studies. Hence, no direct comparisons can be made yet, and therefore, further work is required to understand the precise mechanisms involved and the full extent of possible benefits in any clinical applications which may be developed to improve, enhance, and/or accelerate tendon healing.

## Conclusions

There are several limitations to the present pilot study. First, owing to our focus on histological and histomorphometric examination of repair, only one middle-term evaluation point was used. It remains inconclusive whether or not the effects of using BMSCs, AA, and T_3_ alone, and in every possible treatment combination, lead to short- and long-term structural and functional benefits to tendon healing. Secondly, the effectiveness of AA combined with T_3_ in improving strength and stiffness following the repair of rat tendons requires further analysis.

Despite these considerations, the present study would appear to [14–17] confirm the hypothesis that AA used in combination with T_3_ improves tendon healing in vivo, based upon the best histological score evaluation achieved and on collagen I and III ratio measurement. In the present investigation, for the first time, T_3_ has been utilized for tendon healing in an animal model, and that, apart from our previous in vitro study, no other studies have been made that investigate the activity of T_3_ in tendons. For this reason, our experimental model combining AA with T_3_ and with BMSCs is unique to date. Our results must stimulate two further areas of investigation—on the one hand, into the comprehension of the underlying mechanistic processes of T_3_ in a tendon environment and the associated complex interaction between T_3_, AA, and BMSCs; secondly, our data suggest that AA in combination with T_3_ could be used to develop possible cell-free therapy for improved tendon healing. Furthermore, it will be important to identify optimum delivery methods and doses of AA + T_3_ to improve tendon healing and avoid potential toxicity and, ultimately, to translate this approach into clinical applications in humans.

## Abbreviations

AA: Ascorbic acid
BMSCs: Bone marrow mesenchymal stem cells
COMP: Cartilage oligomeric matrix protein
CTRL: Control group
ECM: Extracellular matrix
FSC: Forward scatter
H&E: Hematoxylin and eosin
r: Rat
SD: Standard deviation
SSC: Side scatter
TH: Thyroid hormones
